# A Rare Indolent Course of Rhinocerebral Mucormycosis

**DOI:** 10.1155/2021/4381254

**Published:** 2021-01-27

**Authors:** Aleksandr Khudyakov, Rafsan Ahmed, Chi Doan Huynh, Amir Dehghani, Zhonghua Li, Michael Rose

**Affiliations:** ^1^SUNY Downstate Internal Medicine, SUNY Downstate Health Sciences University, Brooklyn, NY 11203, USA; ^2^Kings County Department of Pathology, SUNY Downstate Health Sciences University, Brooklyn, NY 11203, USA; ^3^Kings County Internal Medicine, SUNY Downstate Health Sciences University, Brooklyn, NY 11203, USA

## Abstract

Mucormycosis is a highly invasive and rapidly progressing form of fungal infection that can be fatal. The infection usually begins after oral or nasal inhalation of fungal spores and can enter the host through a disrupted mucosa or an extraction wound. The organism becomes pathogenic when the host is in an immunocompromised state. There are several clinical presentations of mucormycosis including rhinocerebral, pulmonary, cutaneous, gastrointestinal, disseminated, and miscellaneous forms. The most common clinical presentation of mucormycosis is the rhinocerebral form which has a high predilection for patients with diabetes and metabolic acidosis. An indolent disease course taking weeks to months of this infection is rare making it difficult to diagnose. Therefore, early detection and prompt treatment with surgical and antifungal therapy are very important in achieving good treatment outcomes.

## 1. Introduction

Mucormycosis (zygomycosis) is an opportunistic fungal infection caused by the fungi belonging to the Mucorales order. The genera most commonly implicated in human infections are *Rhizopus*, *Mucor*, and *Rhizomucor*, followed by *Cunninghamella*, *Lichtheimia*, *Saksenaea*, and *Apophysomyces* [1]. These organisms are ubiquitous in nature and usually found in decaying organic matter and in the soil [2]. Mucormycosis infection involves angioinvasion and is the third most common angioinvasive fungal infection after candidiasis and aspergillosis [3, 4]. Mucormycosis affects immunocompromised individuals, most commonly those having diabetes mellitus (particularly in patients with ketoacidosis). Other predisposing factors for mucormycosis include renal failure, whole organ transplant, immunosuppressive therapy, corticosteroids, cirrhosis, protein-energy malnutrition, burns, malignancies (e.g., lymphomas and leukemias), and acquired immune deficiency syndrome (AIDS) [5]. Most human infections involve oral or nasal inhalation of fungal sporangiospores or direct inoculation of organisms into lacerated skin or disrupted mucosa [6]. The fungus may spread to the paranasal sinuses and subsequently to the orbits, brain, and meninges via invasion of vasculature by hyphae resulting in infarction and necrosis of tissue [7]. It is one of the most rapidly progressive and fulminant forms of fungal infections that is fatal without successful management. Early identification and immediate medical and surgical interventions are necessary to prevent the high morbidity and mortality associated with this infection [8]. Although rare, there are reported cases of an indolent disease course for mucormycosis. Harrill et al. reported a total of 301 cases of rhinocerebral mucormycosis published in the literature, of which 18 were chronic mucormycosis (5.6%) [9–11]. Here, we present a case of rhinocerebral mucormycosis in a diabetic woman with an indolent disease course.

## 2. Case Report

A 46-year-old African American female with a past medical history of hypertension and type 2 diabetes mellitus (T2DM) presented to the emergency department (ED) with chief complain of right facial swelling and pain. Two months prior, the patient had her right upper second bicuspid extraction by oral maxillofacial surgical services (OMFS). Subsequently, the patient had developed a maxillary abscess. Multiple in-office incision and drainage (I&D) procedures with a penrose drain placement were performed followed by a 10-day course of amoxicillin by OMFS. Following her most recent I&D procedure, the patient went home and slept; she woke up with significantly worsened right facial swelling and pain which prompted her to come to the ED at our institution.

**Figure 1:**
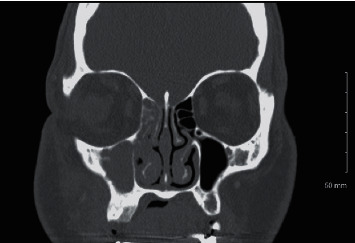
Computed tomography image.

On presentation, her extraoral examination revealed right maxillary and infraorbital swelling which was erythematous and extremely tender to palpation. There was visible left-sided nose deviation and congestion. Cranial nerve examination was remarkable for hypoesthesia in the right V2 distribution. Her intraoral examination revealed no lesions or areas of necrosis. A penrose drain was visible in the gingivobuccal space with no active drainage. Biochemical investigation was significant for white blood cell (WBC) count of 11,530 mm^3^, mild normocytic anemia with hemoglobin of 11.2 g/dL, Na 130 mEq/L, K 6.1 mEq/L, blood urea nitrogen 23 mg/dL, and creatinine 1.15 mg/dL consistent with acute kidney injury (AKI). The patient's initial finger stick was 513 mg/dL, and anion gap was 16. Her glycated hemoglobin was 10.8. Computed tomography (CT) (Figure 1) showed findings consistent with sinusitis with right maxillary sinus opacification. A collection overlying the right maxilla was visible representing a residual abscess. Erosive changes were seen along the walls of the right maxillary sinus secondary to adjacent inflammatory process.

The patient was admitted to general medicine for facial abscess and management of her T2DM. Ear, nose, and throat (ENT) team performed nasal endoscopy which revealed erythematous and inflamed right nasal cavity without evidence of necrosis. The patient was given one-time dose of vancomycin while in the ED and later switched to Unasyn and clindamycin by the medicine team on the first day of admission. On the second day of admission, the patient awoke with increased periorbital edema and pain with decreased range of motion in the right eye. On that day, her physical exam revealed tenderness of the right superior and inferior rim with mild pain on OD supraduction, with no changes in visual acuity. An I&D of infraorbital abscess was performed with 5–7 cc of purulent fluid drained and sent for culture. Subsequently, antibiotic coverage was broadened to vancomycin and Zosyn. The patient remained afebrile with no leukocytosis. Her glucose levels were consistently ranged between 250 and 350 s mg/dL despite continued up titration of insulin.

Preliminary cultures from bedside drainage resulted in growth of *Enterococcus faecalis* and latex-negative *Staphylococcus*, and Unasyn was restarted the fourth day of admission in favor of vancomycin and Zosyn. However, the patient's status worsened over the next 24 hours, and therefore Unasyn was replaced with vancomycin and cefepime, and one dose of Flagyl was administered. Despite broad antibiotic coverage, daily nasal saline irrigation, and mupirocin ointment, her right facial swelling persisted with right periorbital edema and painful abduction. Subsequently, a CT done on day seven of admission showed worsened phlegmonous changes overlying the right maxilla. That day she had a bedside drainage of right nasal ala which was sent for culture.

**Figure 2:**
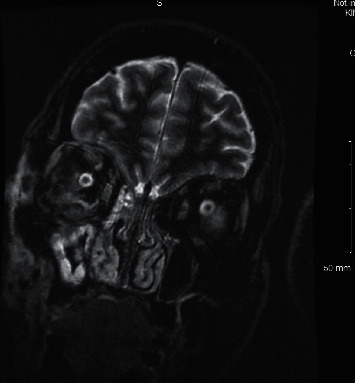
Magnetic resonance imaging.

With the continued management, the patient experienced both subjective and objective improvement of her right facial and orbital swelling; however, the patient started to endorse increased unilateral right temporal headaches. In addition, the patient started to endorse diplopia, and examination was remarkable for impaired right eye abduction and significantly impaired adduction. Subsequently, voriconazole was started on day 9 of admission in favor of antibiotic therapy; however, no improvement was seen with after 36 hours of antifungal treatment. An MRI of the orbits (Figure 2) performed on day ten of admission revealed extensive right nasal, malar, facial, preseptal, and periorbital soft tissue edema consistent with cellulitis and resultant proptosis. Anterior right maxillary sinus wall bony dehiscence and maxillary sinusitis were also evident.

**Figure 3:**
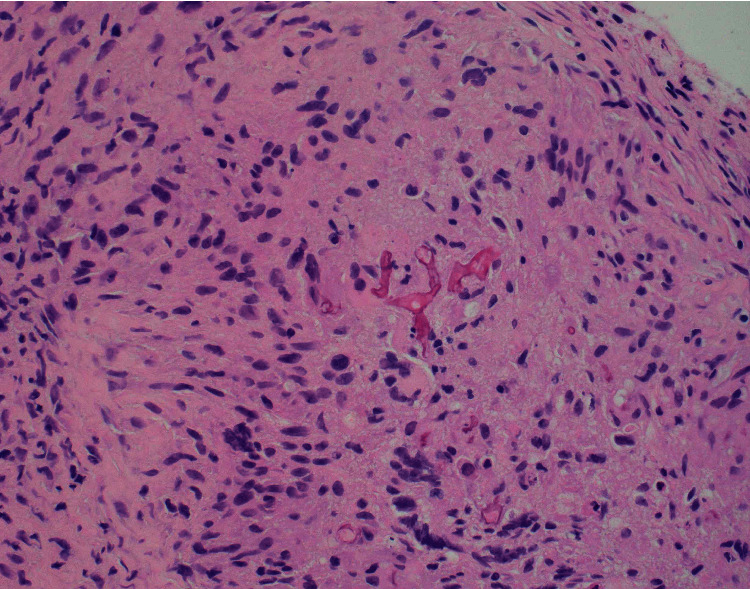
H&E-stained tissue section showing broad aseptate fungal hyphae branching at right angles in a background of inflammatory and epithelial cells.

Pathology of specimens previously obtained from right nasal ala on day seven revealed a possible *Mucor* species. H&E-stained tissue section of nasal mucosa showed broad aseptate fungal hyphae branching at right angles confirming identity as Rhizopus *Mucor* (Figure 3). Based on radiological and histological data, a final diagnosis of invasive mucormycosis was made. As a result, voriconazole was discontinued in favor of caspofungin and amphotericin B lipid complex on day ten of admission. A peripherally inserted central catheter (PICC) line was placed for continued home therapy with amphotericin.

The patient and her family were counseled regarding surgical intervention with possible removal of suspected infective tissue including the right eye and contents of the right orbit to which she was not amenable; as a result, medical management remained the treatment of choice. Intraocular irrigation with amphotericin was also performed by ophthalmology. Treatment with amphotericin B lipid complex was complicated by nausea, vomiting, and AKI, and as a result, it was discontinued in favor of posaconazole. Maxillary cultures from day twenty one came back positive for *Streptococcus* viridans. The patient was started on Zosyn which was later changed to Unasyn on day twenty eight of admission. The patient was started on Decadron 10 mg IV on day twenty five for possible fixed globus according to ophthalmology which was later switched to methylprednisolone 500 mg IV BID on day twenty seven. With the addition of steroids, the patient's facial and orbital swelling showed significant clinical improvement; however, methylprednisolone was discontinued on day thirty due to elevated blood glucose levels. It was discussed with the patient that she can be switched to Ambisome (amphotericin B liposomal complex) from amphotericin B lipid complex as she could not tolerate the latter. Despite recommendations, the patient requested to leave against medical advice and was discharged with home medication of posaconazole 300 mg daily and close outpatient follow-up.

The patient was admitted again the following month with chief complain of painful swollen eyes. She had mucus-like substance in her eyes and was unable to open them and endorsed blurry vision. The patient was admitted to general medicine for management of invasive mucormycosis and was treated with Unasyn and Ambisome. Ophthalmology deemed that no surgical intervention was indicated at the time. Treatment with Ambisome was complicated with nausea, vomiting, and hypokalemia. Complications were managed with pretreatment of intravenous fluids (IV), and she was started on potassium supplements and Zofran with QTc monitoring. She was discharged on Ambisome, potassium tablets, and a 14-day course of Augmentin.

The patient was admitted again on the same month due to chief complain of reduced oral intake and nausea and vomiting. She was unable to tolerate the Ambisome therapy and was found to have AKI, hypokalemia, and hypomagnesemia. Posaconazole was restarted. An MRI of the orbits and paranasal sinuses was done which showed improved inflammatory changes of the right maxillary sinus with no intracranial pathology. Once her electrolytes were within normal limits, the patient was discharged with home medication of posaconazole 300 mg daily. Several outpatient appointments were made, and she was advised to attend them.

## 3. Discussion

Mucormycosis is a highly invasive and rapidly progressing form of fungal infection that is caused by Zygomycetes, a unique class of fungi that reproduce by forming zygospores. The fungi are usually not virulent and can be found ubiquitously in nature. In addition, it can be cultured from the nose, mouth, and throat of healthy individuals. The fungi usually cause infection when host immunity is compromised and is specially seen in individuals with poorly controlled diabetes. When the host is in an immunocompromised state, a mucosal laceration or an extraction wound can be a site of entry of mucormycosis, and both conditions were present in our patient [12].

There are several clinical presentations of mucormycosis including rhinocerebral, pulmonary cutaneous, gastrointestinal, disseminated, and miscellaneous forms [13]. The most common clinical presentation of mucormycosis is the rhinocerebral form which starts by inhalation of spores through the nose with extension to the paranasal sinuses in an immunocompromised host [14]. Diabetes with metabolic acidosis is the most common underlying condition in rhinocerebral mucormycosis [1]. This form of mucormycosis usually presents with fever, facial swelling, headache, sinusitis, sinus pain, nasal congestion, and discharge. In addition, direct spread to surrounding structures, such as the orbits, palate, and brain, usually occurs rapidly within the span of a few days. Involvement of the orbits can result in periorbital edema, proptosis, decreased vision, and ophthalmoplegia. Vascular invasion of the fungus can cause thrombosis, ischemia, and infarction of affected tissues resulting in nasal or palatal ulceration and necrosis, which is a common finding [15]. Radiologically, opacification of sinuses and bony erosion of surrounding sinus walls may be visible. Histopathologically, fungal cultures demonstrate broad aseptate hyphae that branch at right angles [5].

The presentation of mucormycosis in our patient was unique in a few aspects which made it a challenge to establish the diagnosis. The course of the infection was indolent with several weeks of worsening facial swelling and pain and subsequent involvement of the right orbit with associated ophthalmologic symptoms. Most cases of mucormycosis are rapidly progressing; however, chronic presentations of rhinocerebral mucormycosis have been reported. The chronic infection is characterized by a slowly progressing indolent disease course and ophthalmologic symptoms of proptosis, decreased vision, and ophthalmoplegia [9, 10]. Early diagnosis in our patient was made more difficult due to the absence of necrotic tissue. In addition, preliminary cultures grew bacteria in the presence of abscesses, and as a result, a great deal of focus was given towards tailoring antibiotic management.

It has been suggested that early diagnosing and prompt treatment with surgical debridement and antifungal therapy can improve treatment outcomes [14]. It has been shown that combination treatment with surgical debridement and antifungal therapy significantly improves survival in rhinocerebral mucormycosis as opposed to treatment with antifungals alone [16, 17]. The benefits and risks of combination therapy were explained to the patient and her family; however, the patient opted out for surgical management as that may include possible enucleation. As a result, medical management was the treatment of choice. We started the patient on IV amphotericin B lipid complex since it is recommended as the drug of choice for initial therapy for mucormycosis [18]. Caspofungin was also added to her management as it was shown that caspofungin plus amphotericin B lipid complex have synergistic effect [19]. Intraorbital irrigation with amphotericin was also performed to prevent local extension of the infection as outlined in a previous study [20]. The patient responded to our management as her facial and orbital swelling improved significantly, and she regained some motion of her right eye. However, amphotericin B lipid complex was discontinued due to its nephrotoxic effects on the patient. Posaconazole was started as it is recommended as the treatment of choice for patients who do not tolerate or cannot respond to amphotericin B [21]. The patient was discharged against medical advice and was readmitted twice within the following month due to complications of her disease course and medical management.

## 4. Conclusion

Mucormycosis is a highly invasive and rapidly progressing form of fungal infection that can be fatal. Although rare, indolent disease course of this infection has been reported. Here, we described a very rare case of rhinocerebral mucormycosis with an indolent disease course. Absence of tissue necrosis is also an unusual feature of our case. Principles of management for mucormycosis include early disease diagnosis, reversal of predisposing conditions, and prompt surgical and antifungal management. In our case, diagnosis was delayed due to its unusual presentation as well as initial cultures showing bacterial infection in the presence of abscesses. When diagnosis was made, appropriate medical management was initiated; however, the patient refused any surgical intervention. Improvement in the patient's symptoms was observed; however, resolution of the infection was not achieved at the time of writing this paper. Due to the rarity of mucormycosis, particularly the indolent presentation, knowledge and awareness of the disease is emphasized amongst physicians. The importance of early detection and appropriate management with surgical and antifungal combination therapy is also emphasized in our case.
